# Role of Systemic Factors in Improving the Prognosis of Diabetic Retinal Disease and Predicting Response to Diabetic Retinopathy Treatment

**DOI:** 10.1016/j.xops.2024.100494

**Published:** 2024-02-17

**Authors:** Joe Mellor, Anita Jeyam, Joline W.J. Beulens, Sanjeeb Bhandari, Geoffrey Broadhead, Emily Chew, Ward Fickweiler, Amber van der Heijden, Daniel Gordin, Rafael Simó, Janet Snell-Bergeon, Anniina Tynjälä, Helen Colhoun

**Affiliations:** 1 Centre for Population Health Sciences, Usher Institute, University of Edinburgh, Edinburgh, Scotland; 2 Centre for Genomic & Experimental Medicine, Institute of Genetics and Cancer, University of Edinburgh, Western General Hospital Crewe Road, Edinburgh, Scotland; 3 Department of Epidemiology & Data Science, Amsterdam Public Health Research Institute, Amsterdam UMC, location VUmc, Amsterdam, the Netherlands; 4 Division of Epidemiology and Clinical Applications, National Eye Institute, National Institutes of Health, Bethesda, Maryland; 5 Department of General Practice, Amsterdam Public Health Institute, Amsterdam UMC, location VUmc, Amsterdam, the Netherlands; 6 Endocrinology & Nutrition, Institut de Recerca Hospital Universitari Vall d’Hebron (VHIR), Barcelona, Spain; 7 Clinical Epidemiology Division, Barbara Davis Center for Diabetes, University of Colorado Anschutz Medical Campus, Colorado; 8 Folkhälsan Institute of Genetics, Folkhälsan Research Center, Department of Nephrology, Helsinki University Hospital, University of Helsinki, Finland; 9 Beetham Eye Institute, Joslin Diabetes Center, Boston, Massachusetts; 10 Department of Ophthalmology, Harvard Medical School, Boston, Massachusetts

**Keywords:** Biomarkers, Diabetic retinopathy, Prediction, Systemic factors, Systemic health

## Abstract

**Topic:**

To review clinical evidence on systemic factors that might be relevant to update diabetic retinal disease (DRD) staging systems, including prediction of DRD onset, progression, and response to treatment.

**Clinical relevance:**

Systemic factors may improve new staging systems for DRD to better assess risk of disease worsening and predict response to therapy.

**Methods:**

The Systemic Health Working Group of the Mary Tyler Moore Vision Initiative reviewed systemic factors individually and in multivariate models for prediction of DRD onset or progression (i.e., prognosis) or response to treatments (prediction).

**Results:**

There was consistent evidence for associations of longer diabetes duration, higher glycosylated hemoglobin (HbA1c), and male sex with DRD onset and progression. There is strong trial evidence for the effect of reducing HbA1c and reducing DRD progression. There is strong evidence that higher blood pressure (BP) is a risk factor for DRD incidence and for progression. Pregnancy has been consistently reported to be associated with worsening of DRD but recent studies reflecting modern care standards are lacking. In studies examining multivariate prognostic models of DRD onset, HbA1c and diabetes duration were consistently retained as significant predictors of DRD onset. There was evidence of associations of BP and sex with DRD onset. In multivariate prognostic models examining DRD progression, retinal measures were consistently found to be a significant predictor of DRD with little evidence of any useful marginal increment in prognostic information with the inclusion of systemic risk factor data apart from retinal image data in multivariate models. For predicting the impact of treatment, although there are small studies that quantify prognostic information based on imaging data alone or systemic factors alone, there are currently no large studies that quantify marginal prognostic information within a multivariate model, including both imaging and systemic factors.

**Conclusion:**

With standard imaging techniques and ways of processing images rapidly evolving, an international network of centers is needed to routinely capture systemic health factors simultaneously to retinal images so that gains in prediction increment may be precisely quantified to determine the usefulness of various health factors in the prognosis of DRD and prediction of response to treatment.

**Financial Disclosure(s):**

Proprietary or commercial disclosure may be found in the Footnotes and Disclosures at the end of this article.

The systemic factor, glycemia, is well established as an important causal risk factor for the development and progression of diabetic retinal disease (DRD) (DRD is an umbrella term for the aggregation of diabetic retinopathy [DR], diabetic macular edema [DME], and diabetic retinal neurodegeneration which we use to recognize that there is a retinal neurodegenerative component in diabetes that may need to be accounted for in staging, clinical assessment, therapeutics development, and treatment). Optimization of glycemic control, blood pressure (BP), and lipids, are accepted standards of care for prevention of DRD.^1^ Other systemic factors are also likely to play a causal role. Thus, any staging system for DRD needs to consider the potential utility of incorporating data on systemic factors into the grading system.

Current DRD staging systems in widespread use, such as the ETDRS DRD severity scale or the international DRD grading scale, do not incorporate any systemic factor data. In most countries with regular screening systems, such as the United Kingdom (UK), retinal image acquisition with some grading system alone is the basis of the screening system. However, more recently, systemic factors have been incorporated into commercially available prognostic tools on the basis that they may substantially improve prognosis.^2^

The goals of the Mary Tyler Moore Vision Initiative DRD staging update project are to develop a revised, multidimensional DRD severity scale that can be used to better define DRD, stage individual risk of disease worsening, predict and measure response to therapy, and support clinical trials evaluating novel therapies. These goals map broadly onto the United States (US) Food and Drug Administration Biomarkers, EndpointS and other Tools framework that defines diagnostic, monitoring, prognostic, predictive, pharmacodynamic, safety, susceptibility/risk, and surrogate end point biomarkers.

In this paper, our focus was on evaluating the marginal increment in prognostication and prediction provided by systemic factors apart from direct measures of the state of the retina. We considered that regarding the other biomarkers, EndpointS and other Tools biomarker types systemic factors will not play a role as diagnostic, monitoring, or surrogate end point biomarkers in DRD because they would never replace direct end organ (i.e., retinal) measures. Neither are systemic factors direct pharmacodynamic biomarkers of retinal treatments, such as laser or anti-VEGF therapy. Therefore, it is worth reiterating the difference between prognostic and susceptibility/risk biomarkers. The former pertains to those who have some evidence of the disease or medical condition of interest whereas the latter pertains to those who do not currently have clinically apparent disease. In reality, it is often difficult to distinguish these types of biomarkers. Predictive biomarkers predict response (note that the terminology of prognosis and predictive can vary across fields, with prognostic models often called prediction models in e.g., epidemiology studies) to therapy in the context of DRD; therefore, the question is whether systemic factors usefully predict response to laser or anti-VEGF therapy. Note that our focus is on prognosis and prediction, not causation. Biomarkers may be prognostic without being causal and may be causal yet not contribute usefully to prognosis.

Regarding scope, we limited our consideration to systemic factors that are routinely captured in people with diabetes in most clinical settings, such as annual reviews (Box S1, available at www.ophthalmologyscience.org). We did this because only such factors would actually be of use in any practical usage of a new staging system. We conducted a pragmatic review of the literature for individual systemic factors and conducted a more formal systematic review of the literature on multivariate prognostic and prediction models.

## Methods

### Organization

The work described here is part of a wider initiative organized through the Diabetic Retinal Disease Staging System Update Effort, which is a project of the “Mary Tyler Moore Vision Initiative”^3^ and which is supported by the Mary Tyler Moore and S. Robert Levine, MD Charitable Foundation and JDRF. This is one work package, the other topic areas being Vascular Retina, Neural Retina, Basic and Cellular Mechanisms, Visual Function, and Quality of Life. For this work package, we convened an international working group with a track record of research in the epidemiology of diabetes and its complications as listed in the authorship.

### Systemic Factors Explored

As noted earlier, from the outset, we limited our consideration to systemic factors that are routinely captured in people with diabetes in clinical settings as shown in Box S1 (available at www.ophthalmologyscience.org). Routinely captured system factors are those variables that would typically be recorded because they are part of recommendations for the management of diabetes by organizations, such as ADA^4^ and NICE.

### Approach

We conducted a number of pragmatic literature searches. The searches were pragmatic in the sense that although they adhered to the principles of systematic review frameworks, they omitted some formal aspects of the frameworks such as preregistering. The details of searches for individual risk factors, prognostic models, and prediction of therapeutic response to retinal therapies are presented separately.

#### Pragmatic Literature Search on Individual Risk Factors

We assigned specific factors to individual teams of the working group. We asked each team to do a pragmatic literature search and to document the relevant output of this in a standardized Evidence Grid based on the US Food and Drug Administration Biomarker Qualification guidelines rather than a fully documented systematic review of the literature to the Preferred Reporting Items for Systematic Reviews and Meta-Analysis (PRISMA) standards. We did this because the timescale and resources available did not support a PRISMA approach.^5^ The search terms for these individual systemic risk factor literature reviews are given in Methods S1 (available at www.ophthalmologyscience.org). The lead team for each systemic factor agreed and completed a standardized Evidence Grid (see Results S1 section, available at www.ophthalmologyscience.org) and provided a short textual summary of key conclusions with respect to whether consistent associations were found between individual systemic factors and DRD onset or progression. The literature was summarized using a modified biomarker evaluation nomenclature,^6^ with “1A” denoting consistent evidence from randomized controlled trials (RCTs) and observational cohort studies, “1C” denoting consistent evidence from observational cohort studies, “2C” denoting some evidence but inconsistent from observational cohort studies, and “None” denoting no evidence from observational cohort studies or trials as yet. We consider strong evidence to be at level “1A” and moderate evidence to be at level “1C.” We then conducted a more formal search of the literature on the performance of models for prediction of DRD onset or progression (i.e., prognostic models), or both, and on models for prediction of response to treatment (prediction models) as detailed below.

#### Pragmatic Literature Search for Prognostic Models

A recent PRISMA standard systematic review reported studies describing the development of prognostic models for the risk of future retinopathy applicable to people with type 2 diabetes (T2D).^7^ For the current study, similar studies describing retinopathy prognostic models in people with type 1 diabetes (T1D) were added by the author of the original review (A.H.). These also included type 1 studies that had been retrieved from the search strategy of the original PRISMA type 2 systematic review. Studies were considered eligible for inclusion when the population used for model development was followed for ≥ 1 year. The outcomes considered were future incidence and progression of retinopathy. The literature was searched using terms related to retinal disease, prediction models, and diabetes. Data on the type of prognostic factors, model performance in terms of calibration (agreement between predicted and observed retinopathy risk) and discrimination (ability of the model to differentiate between those who will develop retinopathy and those who will not) were extracted from the included studies.

#### Pragmatic Literature Search for Prediction of Therapeutic Response to Retinal Therapies

We performed a systematic search on PubMed on April 18, 2021. Our search process was informed by PRISMA guidelines but was more pragmatic in nature. The primary scope was to capture studies that would quantify predictive information of treatment response for DRD, such as DR and DME. Because the literature was sparse, the query was made wide enough to also include studies reporting associations of factors to therapeutic response. We used the following query: (prognos∗[tiab] OR predict∗[tiab] OR biomarker∗[tiab] OR associa∗[tiab] OR correl∗[tiab]) AND ("vision gain"[tiab] OR "vision improvement"[tiab] OR "predicting response"[tiab]) AND (macul∗[tiab] OR retina∗[tiab] OR retinopathy [tiab]). References from these studies were followed and secondary searches using Google Scholar informed by the results of the primary search were performed. The search strategy is illustrated in Figure S1 (available at www.ophthalmologyscience.org). Studies were considered eligible for inclusion for populations containing people with DR or DME where a therapeutic invention was given. The outcomes considered for inclusion were follow-up visual acuity (VA), retinal thickness, intraocular pressure, DR, and DME or change in those outcomes from baseline.

## Results

Associations can be assumed to be for DRD onset unless explicitly stated.

### Age and Diabetes Duration

As reported in Table 1, there was consistent evidence of a positive association between diabetes duration and DRD. Some studies also reported a positive association between age and DRD, but whether this is an age effect per se, or a diabetes duration effect is unclear.

**Table 1:** Summary of Evidence for Association with Diabetic Retinal Disease from Single-Factor Evaluation

Factor	Evidence for Prospective Association with Incident Diabetic Retinal Disease	Evidence for Prospective Association with Diabetic Retinal Disease Progression	Evidence That it Contributes Predictive Information beyond Retinal Image	Evidence for Role as Predictive of Treatment Response
Age	1C	1C	2C	Not assessed as yet
Diabetes duration	1A	1A	1C	Not assessed as yet
Sex	1C	1C	1C	Not assessed as yet
Diabetes type	1C	1C	2C	Not assessed as yet
Socioeconomic status	1C	1C	2C	Not assessed as yet
Ethnicity	2C	2C	None	Not assessed as yet
HbA1c	1A	1A	1C	Not assessed as yet
Glycemic variability	2C	2C	None	Not assessed as yet
Blood pressure	1A	1A	2C	Not assessed as yet
Blood pressure variability	2C	2C	None	Not assessed as yet
BMI	2C	2C	2C	Not assessed as yet
Lipids	2C	2C	2C	Not assessed as yet
Insulin resistance	2C	2C	None	Not assessed as yet
C-peptide	1C	1C	2C	Not assessed as yet
Smoking	2C	2C	2C	Not assessed as yet
CVD	1C	1C	2C	Not assessed as yet
Neuropathy	2C	2C	None	Not assessed as yet
eGFR/albuminuria/DKD	1C	1C	2C	Not assessed as yet
Pregnancy	1C	1C	1C	Not assessed as yet

Evidence Levels
*1A denotes consistent evidence from randomized controlled trials and observational cohort studies* *1C denotes consistent evidence from observational cohort studies* *2C denotes some evidence butinconsistent from observational cohort studies* *None indicatesno evidence from observational cohort studies or trials as yet*

BMI = body mass index; CVD = cardiovascular disease; DKD = diabetic kidney disease; eGFR = estimated glomerular filtration rate; HbA1c = glycated hemoglobin.

The Wisconsin Epidemiologic Study of Diabetic Retinopathy (WESDR)^8^ reported that, in a cross-sectional study of 1370 people diagnosed with diabetes at ≥ 30 years, severity of diabetes was associated with younger age at diagnosis and longer diabetes duration. In another WESDR study of 996 individuals diagnosed with diabetes at < 30 years,^9^ severity of diabetes was associated with longer diabetes duration. A review of WESDR baseline and 4-year follow-up studies^10^ indicated that longer diabetes duration was associated with a higher 4-year risk of proliferative retinopathy. The Diabetes Control and Complications Trial/Epidemiology of Diabetes Interventions and Complications (DCCT/EDIC) study of people with T1D also reported that a higher diabetes duration was associated with a higher risk of proliferative DR (PDR)^11^ (hazard ratio [HR] =1.15 [1.12, 1.18]). A cross-sectional registry-based study in Sweden^12^ of 2232 people with diabetes drew similar conclusions regarding the association between diabetes duration and DRD. In individuals with T1D from the prospective German Diabetes Documentation System survey,^13^ diabetes duration was associated with increased odds of both any and advanced retinopathy (respectively, odds ratio [OR] 1.167 per year and 1.124 per year). Adding to these, the Los Angeles Latino Eye Study (LALES) study^14^ found that, in individuals with T2D, a longer duration of known diabetes was associated with higher odds of any DRD (OR = 1.08 per year), and of PDR (1.06 per year).

Similar findings have been observed in the UK. Scanlon et al’s^15^ study of retinopathy screening attendance in the UK reported that diabetes duration was associated with both higher odds of referable retinopathy (OR ranging from 3.5 [2.8–4.5] for 5–9 years duration to 15.8 [12.3–20.4] for ≥ 20 years vs. ≤ 5 years) and higher odds of urgent referral to retinopathy (OR ranging from 4.5 [2.5–8.1] for 5–9 years duration to 33 (20–54) for ≥ 20 years vs. ≤ 5 years); while in the UK Prospective Diabetes Study (UKPDS) 50, Stratton et al^16^ reported that older age was associated with a higher relative risk of DR progression in people with T2D. The Chennai Urban Rural Epidemiology Study in a South Indian population with T2D^17^ (N = 1715) showed that older age (OR = 1.20 [1.08, 1.33] per 10 years) and duration of diabetes (OR = 1.65 [1.50, 1.82] per 5 years) were both significantly associated with DR severity.

### Sex

Several studies have consistently reported that male sex is associated with DRD.

A WESDR study of individuals diagnosed with diabetes at < 30 years^9^ reported that severity of DR was associated with male sex in those with diabetes duration of > 10 years.

Scanlon et al^15^ reported that the female sex was associated with lower odds of referable retinopathy compared with the male sex (OR = 0.82 [0.72–0.93]); whereas in UKPDS 50, Stratton et al^16^ reported that, in people with T2D, the incidence of retinopathy was similar for males and females but female versus male sex was associated with a lower relative risk of DR progression. Finally, the LALES study^14^ found that the male sex was associated with higher odds of any DRD (OR = 1.5) and the male sex was significantly associated with DR severity (OR = 1.49 [1.17, 1.90]) in the Chennai Urban Rural Epidemiology Study.^17^

### Type of Diabetes

Several studies have reported that the incidence of retinopathy and its progression is higher in type 1 than in T2D. In Scotland, T1D was a positive risk factor for referral due to PDR even after adjustment for age, sex and diabetes duration, and screening method (OR = 1.35; OR, 1.06–.73, *P* = 0.017).^18^^,^^19^ In other studies in which multivariable models for DRD have assessed the type of diabetes as a prognostic factor, T1D was associated with higher DRD incidence (risk ratios [RRs], 1.4–2.4) in 2 studies^20^^,^^21^ but not in another.^22^ However, although these studies have adjusted for diabetes duration and other risk factors, such adjustment is limited by the lack of overlap in diabetes prevalence by type at different ages. One study suggests that T1D has higher risks than T2D among youth, but whether even restricted to youths this is accounted for by earlier onset and worse glycemic control in youth with T1D remains possible. Wang et al^23^ reported that among 2240 youth with T1D and 1768 youth with T2D, 20.1% and 7.2% developed DR, over a median follow-up of 3.2 and 3.1 years, respectively. Survival curves demonstrated that youth with T1D developed DR faster than youth with T2D (*P* < 0.0001). However, analysis of the SEARCH for Diabetes in Youth registry study concluded that the age-adjusted prevalence of retinopathy was higher in youth onset T2D (9.1%) compared with T1D (5.6%) during teenage years and young adulthood.^24^ Further, an analysis of the Canadian Diabetes Education Resource for Children and Adolescents found a similar prevalence of retinopathy among those with T1D in youth (13.8%) and T2D in youth (11.7%).^25^

Most studies therefore find type of diabetes to be a useful prognostic factor for DRD, although whether type itself or other confounding factors explain this is unclear. For instance, there is increasing evidence that increasing the use of technology such as insulin pumps lowers the risk of DR for people with T1D (see Technology Use for Managing Glycemia section). At present, however, most DRD screening programs do not offer separate intervals for screening by type of diabetes for any given current level of DRD.

### Socioeconomic Status

Socioeconomic status (SES) encompasses several interrelated parameters, including income, education, neighborhood, insurance status, and employment. Socioeconomic status can be assessed through direct queries regarding factors such as income, employment status, education, and insurance through questionnaires. In addition, neighborhood information can be assessed using Geographic Information Systems, and composite “deprivation index” scores.

Lower SES has consistently been linked to lower attendance at DRD screenings and is also associated with risk factors for retinopathy that include higher glycosylated hemoglobin (HbA1c), increased smoking prevalence, and higher levels of cholesterol. The relationship between SES and DRD prevalence may be stronger in T1D than in T2D. For example, a cross-sectional study of 1861 individuals with T1D and 18 197 individuals with T2D diabetes found a significant association between the lowest SES according to the Scottish Index of Multiple Deprivation and any DR in people with T1D (OR, 2.4; 95% confidence interval [CI], 1.36–4.27; *P* = 0.002), but not in people with T2D (OR, 0.85; 95% CI, 0.71–1.02).^26^ However, other studies have shown an association between DR and SES in youth onset T2D.^27^

In a 9-year prospective study of 150 people with T1D, the risk of developing retinopathy was increased almost threefold (HR, 2.95; 95% CI, 1.08–8.0) in residents of lower SES areas and diagnosed as adults compared with people with childhood onset and living in areas with higher SES.^28^ In a study among adults with diabetes in Indonesia,^29^ almost 85% had not had an eye examination in the last year. In this study, lack of knowledge was the primary reason for not undergoing regular eye examinations, with financial barriers only reported by 13.8% of subjects.

### Ethnicity

There is some evidence for variation in the risk of DRD based on ethnicity (e.g., higher rates in Hispanic and Black populations in the US), but it is not yet known if this variation relates to ethnicity specifically or to other factors that may differ between ethnicities, such as glycemic control, medication compliance, and diet. In the UK, a study of the prevalence of DR or maculopathy in those attending screening demonstrated a higher rate in the ethnic minorities that included South Asians and African/Afro-Caribbeans. However, this study did not adjust for the risk factors of glycemic control, duration of diabetes, hypertension, and smoking status.^30^ In another study, the risk of DR subtypes in Indian migrants to the UK more closely resembled the risk of other UK ethnicities than the risk in India, suggesting it is not ethnicity alone that is responsible for the variation in DR risk.^31^ Many studies have found no association between ethnicity and DRD once factors potentially differing between ethnicities are accounted for.^32^, ^33^, ^34^ Also, many studies that examined ethnicity and retinopathy have not adjusted for other risk factors, such as duration of disease or glucose levels. Even those few papers that have adjusted for multiple confounding factors often did not investigate whether differential HbA1c thresholds may exist depending on ethnicity, or found conflicting results.

An example from the Atherosclerosis Risk in Communities^33^ of 10 363 participants demonstrated the results of studies of testing for interactions of biomarkers with race in models of incident outcomes including DR. These biomarkers included HbA1c, fructosamine, glycated albumin, and 1,5-anhydroglucitol (1,5 A-G). The median values of biomarkers were higher among Black versus White people (all *P* < 0.001) but the ORs for each biomarker with prevalent retinopathy were similar by race (all *P* values for interaction by race > 0.10).

The Singapore National Health Survey^35^ and the Singapore Prospective Study Program found variations in diabetes prevalence between Indian, Malay and Chinese people (see Results S1, available at www.ophthalmologyscience.org). Although the prevalence of any DR and any DME was higher in Indian versus Chinese and Malay people, the major risk factors for DRD were similar across the 3 ethnic groups.

In South Africa,^36^ prevalence of any DRD was higher for Asian Indians and indigenous Africans compared with White people. However, increasing duration of diabetes and poor glycemic control were the strongest risk factors associated with any and referable DRD in both type 1 and T2D.

Wong et al^32^ found a higher prevalence of DRD in Black and Hispanic populations in the Multi-ethnic Study of Atherosclerosis Data (*P* = 0.02 and *P* = 0.007, comparing racial/ethnic differences for retinopathy and DME, respectively; more details in Results S1, available at www.ophthalmologyscience.org), but also found that race was not an independent prognostic factor of retinopathy once other factors (duration or diabetes, fasting glucose, and use of diabetic medications) were accounted for. Significant prognostic factors of any retinopathy were longer duration of diabetes, higher fasting serum glucose, use of diabetic oral medication or insulin, and greater waist–hip ratio. The most recent publication from an American cohort on this topic^37^ suggested that there may be differing HbA1c thresholds for retinopathy. Overall, the threshold was 6.0%, but thresholds differed by race/ethnicity (e.g., Hispanic people 6.4%, non-Hispanic Black people 6.5%, and non-Hispanic White people 6.0%).

In the LALES, Native American ancestry in Latino T2DM subjects was a risk factor for severe DR (multivariable-adjusted OR, 1.87; *P* = 0.002) independently of other risk factors.^38^

### Glycemia

#### HbA1c

The most relevant risk factor for the development of DRD is poor glycemic control as measured by higher HbA1c. As reported in Table 1, there is robust evidence regarding the relationship between blood glucose levels and the development and progression of DRD. Intensive versus conventional glycemic management was associated with a 39% reduction in the risk of laser photocoagulation in the population with T2D of the UKPDS.^39^ Tight versus less tight glycemic control in the type 1 diabetic population of the DCCT reduced the risk of new retinopathy by 76% and of the progression of existing retinopathy by 54%.^40^ Recently, it has been reported that keeping the HbA1c level < 7.6% (60 mmol/mol) as a treatment target seems to prevent PDR for up to 20 years in people with T1D.^41^

There is no information regarding the long-term effect of blood glucose control on retinal neurodysfunction or neurodegeneration.

#### Metrics

In the UKPDS, for every 1% decrease in HbA1c, there was a reduction in 40% of DRD development, 25% progression to vision-threatening DR, 25% need for laser therapy, and 15% blindness in people with diabetes.^42^

In the DCCT, a 10% lower HbA1c (e.g., 8% vs. 7.2%) was associated with a 43% lower risk of DR progression in the intensive group and a 45% lower risk in the conventional group. These risk gradients applied over the observed range of HbA1c values and were unaffected by adjustment for other covariates.^43^

Diabetes duration is even more important than HbA1c as a risk factor for the development and progression of DRD. For instance, a study using NHANES showed an OR of 8.51 (95% CI, 3.70–19.54) for the association between diabetes duration and DME compared with an OR of 1.47 (95% CI, 1.26–1.71) for HbA1c.^44^ Therefore, diabetes duration should be taken into account when evaluating the impact of HbA1c on DRD. Indeed, the PDR risk associated with a 1% point increase in HbA1c was equivalent to the risk associated with 6.4 (95% CI, 5.3–7.4) additional years' duration of T1D.^45^

#### Metabolic Memory

The phenomenon of ongoing beneficial effects on diabetic complications after a period of improved glycemic control even if followed by a return to usual (often poorer) metabolic control has been described as “metabolic memory” or “legacy effect.” This phenomenon has been observed in the EDIC study, an observational follow-up of DCCT participants.^46^ Over the first 4 years of EDIC, the former DCCT intensive therapy group experienced a lower incidence of further progression of DRD than did the former conventional group, despite similar HbA1c levels in both groups.^47^ This “metabolic memory” persisted after 10 years, even having a lesser degree of metabolic control.^48^ By year 18 of EDIC, the effect of “metabolic memory” had largely faded, with no further divergence of retinopathy rates, but the former intensive group continued to have fewer ocular complications, including a substantially lower risk of advanced retinopathy outcomes.^40^ The “legacy effect” has been also observed in people with T2D.^49^, ^50^, ^51^

#### Problems with the Rapid Lowering of HbA1c: Early Worsening of DRD

The therapeutic goal of tight metabolic control should be balanced against the risk of hypoglycemia, especially in older people, in whom aggressive glycemic control does not further reduce retinopathy risk and might even be associated with increased mortality. There is a general consensus for glycemic goals (HbA1c) to take into account age (i.e., children and older adults), risk of hypoglycemic episodes, and concomitant conditions (i.e., comorbidities and pregnancy).^52^

Although there is no doubt regarding the relationship between glycemic control and the long-term development and progression of DRD, initial worsening of DRD has been reported as a consequence of rapid improvement of hyperglycemia. An early worsening was observed in 13.1% of 711 patients with T1D assigned to intensive treatment in DCCT at 6 or 12 months, or both, in comparison with 7.6% of 728 patients assigned to conventional treatment (OR, 2.06; *P* < 0.001).^53^ The most important risk factors for early worsening were a higher HbA1c level at screening, a large reduction of HbA1c (> 2%), and the severity of DRD at baseline.^53^, ^54^, ^55^ A similar phenomenon was reported in patients with T2D after a rapid improvement of blood glucose levels when they were changed from oral agents or diet alone to insulin therapy,^56^^,^^57^ and more recently after bariatric surgery.^58^ As reported in patients with T1D, the magnitude of the reduction of HbA1c and the presence of preexistent DRD were the main factors involved in the risk of this transient or permanent progression of DRD.

More recently, systematic reviews have confirmed this concept and suggested that early worsening of DRD could be particularly relevant when HbA1c is reduced by > 1.5 % in 3 months or > 2% in 6 months.^59^^,^^60^

#### Glycemic Variability

For patients prone to glycemic variability, especially patients with T1D or T2D with severe insulin deficiency, glycemic control is best evaluated by the combination of results from self-monitored blood glucose or continuous glucose monitoring (CGM) and several measurements of HbA1c per year.

Extensive statistical analyses from the DCCT cohort show that “total glycemic exposure” (HbA1c levels over time) accounts for only about 11% of the reduction in microvascular complications.^61^ Plasma glucose variability is an important reason accounting for the weaknesses of the HbA1c level in predicting the development and progression of DRD. The introduction of CGM, which captures the glucose profile over a number of days, has provided an opportunity to develop metrics of glycemic control that deliver valuable information beyond that furnished by the HbA1c level. Among the metrics generated from CGM, time in range (TIR) refers to the time an individual spends within their target glucose range (usually 3.9–10.0 mmol/L), which provides valuable information about whether the frequency and duration of hypoglycemia or hyperglycemia improve over time. In a recent study, TIR was found to be associated with all stages of DR after controlling for age, sex, body mass index (BMI), diabetes duration, BP, lipid profile, and HbA1c level.^62^ In addition, a systematic review and meta-analysis has shown that fasting plasma glucose variability was strongly associated with an increased risk of retinopathy.^63^ Glycosylated hemoglobin variability, which reflects longer-term glucose variability, also contributed to the risk of DRD in type 1 and T2D.^64^, ^65^, ^66^

#### Differential Impact of Antidiabetic Agents on DRD

The strong relationship between the reduction of HbA1c and the beneficial effects on DRD has obscured the necessity of performing clinical trials to investigate the effect of drugs that lower glucose on DRD per se, independent of their effectiveness in lowering blood glucose levels. The lack of studies that address this specific question means that we lack clear information on this issue. Nevertheless, several meta-analyses and real-world population-based studies did not find that dipeptidyl peptidase-4 inhibitors, glucagon-like peptide-1 receptor agonists (GLP-1RA), and sodium-glucose transporter-2 inhibitors have a higher risk of DRD than placebo.^67^, ^68^, ^69^ By contrast, some evidence suggests that sulfonylureas may be associated with an increased risk of DRD.^68^

The reported pleiotropic actions of GLP-1RA in experimental models of DRD, apart from their capacity in lowering blood glucose levels, confers on these drugs a potential extra value in preventing the development or arresting the progression of DRD.^70^ The results obtained in the SUSTAIN-6 study are one exception to this statement. In this study, semaglutide (a long-acting GLP-1R agonist) showed an unexpectedly higher rate of severe DR complications (i.e., vitreous hemorrhage, blindness, or conditions requiring treatment with an intravitreal agent or photocoagulation).^71^ Although this effect was observed in a very small percentage of patients allocated to semaglutide (n = 50 [3%]) vs. (29 [1.8%]), patients of the placebo group, it (HR, 1.76; 95% CI, 1.11–2.78; *P* = 0.02) was a surprising finding because, as mentioned earlier, beneficial rather than deleterious effects on the development of DRD were reported in preclinical studies by using GLP-1RA. In addition, several cohort studies^72^^,^^73^ and meta-analyses^68^^,^^74^ have shown that GLP-1RA did not increase the risk of development and progression of DRD. In addition, 2 independent reports of the US Food and Drug Administration Adverse Reporting System on this issue support this conclusion.^73^^,^^75^ The main reason accounting for this deleterious effect was the rapid decrease in HbA1c levels and the lack of grading of DRD at study entry.^70^^,^^76^ However, a large clinical trial (FOCUS study, NCT03811561) with 4 years of follow-up to further clarify this issue is ongoing.

#### Technology Use for Managing Glycemia

The use of diabetes technologies such as continuous subcutaneous insulin infusion (CSII), CGM, and hybrid closed-loop (HCL) systems has led to improvements in glycemic control.^77^ Poor glycemic control is one of the strongest risk factors for microvascular complications, and both hyperglycemia and glycemic variability may contribute to DRD. The use of CGM has been shown to reduce HbA1c dramatically when started within the first 6 months of diagnosis of T1D,^78^ and HCL systems improve glycemic control as well as variability.^77^

There is biological plausibility that the use of CSII, accompanied by a reduction in blood glucose levels, or a reduction of glycemic variability, could reduce the risk of DRD, as CSII use was associated with greater vascular density in the retina, and lower vascular density may be associated with the development of DR.^79^ In a prospective study of CSII use among 157 people with T1D, over a 4-year follow-up period, there was a significant reduction in HbA1c (from 8.4% ± 1.3% to 7.7% ± 1.3%), but the incidence of DR was low (3.6 per 100 patient-years) and there was no association between baseline HbA1c or change in HbA1c and incidence of retinopathy.^80^ In a small study of 35 people with T1D and 33 people with T2D, associations between glucose parameters measured by CGM and DR were examined cross-sectionally.^81^ There were significant but modest associations between the prevalence of DR and parameters of glycemic variability (glucose standard deviation [OR, 1.03; 95% CI, 1.01–1.06], continuous overlapping net glycemic action calculated every 2 hours during the monitoring period [OR, 1.02; 95% CI, 1.00–1.04]) as well as hyperglycemia (high blood glucose index [OR, 1.1; 95% CI, 1.01–1.18]).^81^ In a larger study of 3262 people with T2D, glucose TIR, and glucose variability parameters were examined in association with mild DRD, moderate DRD, and vision-threatening DR.^62^ Lower TIR and higher glucose variability were associated with a higher grade of DRD, and in multinomial logistic regression, TIR was significantly associated with increased risk of mild, moderate, and vision-threatening DR, independent of glycemic control, BP, lipids, obesity, and demographics.^62^

### Hypertension

The consensus cut-off to define high BP is ≥ 140/90 mmHg. However, targets for treatment must be individualized, taking into account the presence of cardiovascular or its estimated risk at 10 years.^82^ Hypertension is a risk factor for DRD in patients with T2D and T1D as demonstrated in UKDPS^16^^,^^83^ and DCCT/EDICT studies,^11^ respectively. In the DCCT/EDIC study of people with T1D using a multivariable-adjusted model, higher mean diastolic BP (DBP) was associated with incident PDR (HR, 1.0448 per 1 mmHg [95% CI, 1.0255–1.0645], *P* < 0.0001). In the WESDR, higher systolic BP (SBP) was associated with incident DME (HR per 10 mmHg 1.15, 95% CI, 1.04–1.26; *P* = 0.004).^84^ Higher DBP was independently associated with the progression of retinopathy.^85^ The LALES study found an OR of 1.26 (*P* = 0.002) for every 20 mmHg increase in BP.^14^ The Hoorn study estimated that patients with hypertension had more than double the risk of developing retinopathy after 10 years when compared with patients with diabetes with normal BP.^86^

The UKPDS was the first RCT that showed the importance of tight BP control in reducing DR.^16^ A total of 1048 hypertensive people with T2D were randomized into intensive BP control (target SBP/DBP: < 150/85 mmHg) versus the conventional control group (target SBP/DBP: < 180/< 105 mmHg). After 9 years of follow-up, patients with tight BP control had a reduction in risk of DR progression by 34% (99% CI, 11–50) and VA deterioration by 47% (99% CI, 7–70). However, a more recent study performed in a type 2 diabetic population (the Action to Control Cardiovascular Risk in Diabetes [ACCORD-EYE] study) did not reveal any significant effect of intensive BP versus standard treatment in retinopathy progression of ≥3 steps on the ETDRS scale.^51^^,^^87^ However, in this study the intensive treatment arm targeted SBP < 120 mmHg, and the standard treatment arm SBP < 140 mmHg. A Cochrane systematic review conducted in 2015 found 15 trials, including the ACCORD-EYE study, that examined the effect of antihypertensive treatment on retinopathy (5 in T1D and 10 in T2D). It concluded that BP control reduced the incidence of DR (estimated RR 0.80; 95% CI, 0.71–0.92) although effects on progression were only significant for T2D.^88^

Another systematic review from 2015 that captured mostly the same trials of BP lowering, but restricted to people with T2D, reported a relative risk of 0.87 [95% CI, 0.76–0.99] for 3 or more step progression of retinopathy per 10 mmHg-lower SBP.^89^

#### Metrics

It has been shown that every 10 mmHg increase in SBP was associated with 10% increased risk of early DR and 15% risk of PDR or DME.^84^^,^^90^ In the UKDPS study, a 10 mmHg reduction in systolic was associated with approximately 40% to 50% reduction in DRD progression, need for laser treatment, and vision loss.^39^^,^^42^ The systematic review above reported a relative risk of 0.87 (95% CI, 0.76–0.99) for ≥3 step progression of retinopathy per 10 mmHg-lower SBP in T2D.

#### Time Relationship

The deleterious effect can be seen after several years of hypertension, but there are no studies that specifically address this question. A “legacy” effect that occurs with glycemic control has not been reported with hypertension.

#### Ambulatory BP Monitoring

Compared with measurements taken in the clinic setting, ambulatory BP monitoring may provide a better estimate of an individual’s average BP.^91^ Ambulatory BP monitoring phenotypes included daytime, sustained, nocturnal, and isolated nocturnal hypertension, a nondipping BP pattern, and white coat, masked, and masked isolated nocturnal hypertension.^42^ Several of these phenotypes, including elevated mean 24-hour BP, elevated nighttime BP, and nondipping BP pattern have been associated with increased cardiovascular disease (CVD) risk.^91^, ^92^, ^93^, ^94^ However, no prospective data regarding the BP phenotypes and the risk of DRD have been reported. Therefore, clinical trials including ambulatory BP monitoring would be very useful to further determine the role of hypertension and its distinct patterns on the development and progression of DRD.

#### Differential Impact of Antihypertensive Drugs on DRD

It seems that the antihypertensive medications that target the renin-angiotensin system, including angiotensin II receptor antagonists^95^, ^96^, ^97^ and angiotensin-converting-enzyme inhibitors, such as enalapril,^97^ may have additional benefits in slowing DR progression, independent of their hypotensive properties. However, there is weak clinical evidence on this issue, and it seems that the reduction of BP is the key point rather than the type of antihypertensive drug.

### BMI

Results on the association of BMI and DRD have been generally inconsistent. In a cross-sectional study of individuals with diabetes in the Singapore Malay Eye study, higher BMI was associated with a lower prevalence of DRD, with the highest quartiles of BMI being associated with lower odds of both any DRD (OR, 0.5; 95% CI, 0.3–0.7), and moderate DRD (OR, 0.4; 95% CI, 0.2–0.7). Similarly, the WESDR study reported that the severity of DR was associated with smaller body mass in individuals aged ≥ 30 years at diagnosis.^8^ On the other hand, the Hoorn study^98^ reported that increased BMI was positively associated with retinopathy prevalence, with the DCCT^99^ reporting similar results (OR, 1.11).

### Insulin Resistance

Insulin resistance (IR) is observed in type 1 and, more frequently, in T2D, although the mechanisms may differ by type of diabetes. Among patients with T2D, IR often results from obesity and physical inactivity and plays a role in the development of diabetes. Among patients with T1D, obesity and physical activity are similar to the general population,^100^ yet insulin resistance is a common finding in these patients.^101^ Higher levels of IR and IR-related factors have been associated with incident DR in several prospective studies of adults with T1D, including in the EURODIAB Prospective Complications Study^102^ and the Coronary Artery Calcification in Type 1 Diabetes study.^103^ In the EURODIAB Prospective Complications Study, incident DR occurred in 56% of people with T1D followed over 7 years, and fasting triglycerides (OR, 1.24; 95% CI, 1.01–1.54; *P* = 0.04) and waist-to-hip ratio (OR, 1.32; 95% CI, 1.07–1.63; *P* = 0.01) were important predictors of retinopathy incidence, independent of diabetes duration and glucose control. In the Coronary Artery Calcification in Type 1 study, higher estimated insulin sensitivity was associated with > 30% reduced risk of incident self-reported retinopathy (OR, 0.69; 95% CI, 0.50–0.95; *P* = 0.02).^103^ In addition, a case-control study of IR measured by a hyperinsulinemic-euglycemic clamp in patients with T2D found that a higher glucose disposal rate (indicating greater insulin sensitivity) was associated with a 30% reduced risk of PDR.^104^ A study of 70 Japanese people with T2D also showed an association between DR and IR (measured by the HOMA index).^105^ Overall, IR seems to be an important factor related to increased DRD incidence and progression in both type 1 and T2D.

### Lipids

Studies have shown inconsistent results on the associations of lipids and DRD.^106^ A study by Wong et al^107^ (Singapore Malay Eye Study) reported a protective effect of increased total cholesterol. A recent study from Italy by Sasso et al^108^ reported that higher high-density lipoprotein (HDL) cholesterol is a risk factor for PDR.

One of the early studies came from the ETDRS papers^109^ in which serum levels were evaluated for progression to DME with marked retinal hard exudates. In this study, higher total, low-density lipoprotein and very low-density lipoprotein cholesterol were associated with more exudates but there was no significant relationship with triglycerides or with HDL cholesterol. Other ETDRS studies^110^ also showed that elevated serum levels of triglycerides were associated with an increased risk of progression to PDR. For triglycerides levels > 160 mg/dL, the OR was 1.25 (1.06–1.42), *P* = 0.0065.

The DCCT/EDIC cohort of people with T1D showed a positive association between the severity of DR and increased triglyceride levels and a negative association with HDL cholesterol levels.^80^

Klein et al’s WESDR^111^ study showed a significant trend for increasing severity of DR and of retinal hard exudate with increasing cholesterol in insulin-using persons. Cholesterol levels were not related to the severity of either ocular condition in older onset patients. High-density lipoprotein cholesterol was unrelated to the severity of either lesion. In multiple logistic regression analyses, cholesterol was not a significant factor in describing the severity of retinopathy in any group but was a significant factor in describing the severity of retinal hard exudate. Glycosylated hemoglobin and DBP were significant descriptors of the severity of DR in younger-onset patients in these multivariate analyses. Diastolic blood pressure added significantly to explaining the severity of hard exudate in older onset insulin users.

Post hoc analyses of data from the Veterans Affairs Diabetes Trial^74^ of intensive versus standard glycemic control showed that there were interactions between initial and follow-up lipid levels and the effect of intensive glucose lowering on retinopathy. These interactions were not in a consistent direction for total cholesterol but higher HDL cholesterol and lower triglycerides were consistently associated with better response to intensive therapy throughout the study.

A few small-sample studies have reported the serum apolipoprotein ratio (apoB/apoA-1) as a risk factor for DRD^112^^,^^113^ in both T1D and T2D, but this relationship still needs to be better understood. Fort et al^114^ reported a graded decrease in circulating long-chain acylcarnitines and a graded increase in the intermediate-length saturated and monounsaturated triacylglycerols from no DRD to moderate nonproliferative DR (NPDR) independent of HbA1c in persons with T2D. These findings require further investigation.

#### Lipid-Lowering Drugs

##### Fenofibrate

Two RCTs, the Fenofibrate Intervention and Event Lowering in Diabetes (FIELD) and the ACCORD-EYE study,^115^^,^^116^ have reported that fewer patients on fenofibrate had 2 to 3 steps of DRD progression than those on placebo. The ACCORD-EYE study did not evaluate the treatment effect for delaying the onset of DR, whereas FIELD reported similar rates of occurrence of new retinopathy in both the placebo and the fenofibrate arm. In the ACCORD-EYE study of 2856 participants with T2D, a subset (n = 1263) of participants were randomized to fenofibrate 160 mg plus simvastatin versus placebo with simvastatin daily. The results showed a beneficial effect of fenofibrate plus simvastatin for reducing the risk of progression of DR by 3 steps on a person scale or progression to vitrectomy or laser for PDR when compared with placebo. The progression rate of DR was 6.5% with fenofibrate for intensive dyslipidemia therapy, versus 10.2% with placebo (adjusted OR, 0.60; 95% CI, 0.42–0.87; *P* = 0.006).^116^

The FIELD study was a multinational RCT of 9795 patients aged 50 to 75 years with T2D mellitus. A tertiary end point of treatment with laser for DME and PDR was collected as self-reports. Fundus photography was performed on 1012 patients.^115^ The requirement for first laser treatment for all retinopathy was significantly lower in the fenofibrate group than in the placebo group (164 [3.4%] patients on fenofibrate vs. 238 [4.9%] on placebo; HR, 0.69; 95% CI, 0.56–0.84; *P* = 0.0002; absolute risk reduction 1.5% [0.7–2.3]). In the ophthalmology substudy, the primary end point of the 2-step progression of retinopathy grade did not differ significantly between the 2 groups overall (46 [9.6%] patients on fenofibrate vs. 57 [12.3%] on placebo; *P* = 0.19) or in the subset of patients without preexisting retinopathy (43 [11.4%] vs. 43 [11.7%]; *P* = 0.87). By contrast, in patients with preexisting retinopathy, significantly fewer patients on fenofibrate had a 2-step progression than did those on placebo (3 [3.1%] patients vs. 14 [14.6%]; *P* = 0.004). An exploratory composite end point of 2-step progression of retinopathy grade, DME, or laser treatments was significantly lower in the fenofibrate group than in the placebo group (HR, 0.66; 95% CI, 0.47–0.94; *P* = 0.022). The FIELD and ACCORD-EYE studies indicated that the fenofibrate-associated reductions in DRD progression were independent of lipid levels.^117^

The MacuFen Study, a double-masked RCT, evaluated fenofibrates in 110 DME participants not requiring immediate photocoagulation or intraocular treatment and who had adequate BP and diabetes control, was underpowered to detect the benefit of fenofibrate over placebo^118^ (See Results S1 for more details, available at www.ophthalmologyscience.org).

A double-masked, randomized, placebo-controlled study that investigated the safety and efficacy of etofibrate in 296 patients with T2D who had NPDR reported that the diabetic fundus changes improved or remained stable at 12 months from the baseline in the majority of patients in the fibrate group than in the placebo group. Patients in the fibrate group had a significant decrease in the total cholesterol, lipid, and blood glucose levels compared with the placebo group.^119^

##### Statins

A few clinical trials have evaluated the effects of statins in DRD.^120^, ^121^, ^122^ The mean change in VA, DME, and resolution of hard exudates at 6 months was similar in the statin and placebo groups when participants had a normal lipid profile at baseline.^122^ Total cholesterol and low-density lipoproteins at 6 months reduced whereas HDLs increased when patients with NPDR and a raised lipid profile at baseline received statin treatment versus those on placebo.^120^^,^^121^ Hard exudates reduced significantly in the statin group.^121^ More patients in the placebo group had worsening of VA and color fundus images than those in the statin group.^120^ These clinical trials, however, had a small-sample size (30–50) and a short follow-up (6 months).

### Smoking

Smoking is a risk factor for several vascular complications of diabetes, especially macrovascular disease.^123^ The evidence for a link between smoking and microvascular disease is less clear, but there is a biological mechanism through which nicotine can damage retinal vessels and contribute to DRD.^124^^,^^125^ However, evidence from human studies demonstrating associations between smoking and microvascular complications of diabetes, including DRD, is inconclusive.^126^, ^127^, ^128^, ^129^

A large prospective study of DRD, the WESDR study, examined 1210 people with T1D and 1780 people with older onset diabetes.^130^ In this cohort, pack-years of smoking had a univariate association with progression to PDR in older onset diabetes, but this relationship became nonsignificant after adjustment for risk factors.^130^ No strong association was observed between smoking and DR in people with T1D, though there was a trend toward significance between smoking and incidence of retinopathy among people with T1D (*P* value 0.052), leading to the conclusion that smoking is not likely to be an important risk factor for DRD.^130^ Similarly, in a study of 201 patients with T1D, smoking was not associated with PDR over 25 years.^129^ However, in the EURODIAB study, there was a relatively high prevalence of smoking (35% of men and 29% of women were current smokers), and in this cross-sectional analysis of 3250 people with T1D aged 15 to 60 years, there was a modest association between current smoking and worse glycemic control, as well as between smoking and DR.^127^ A meta-analysis of 19 studies including people with T1D and 56 studies of people with T2D showed a significantly increased risk of incidence and prevalence of DRD (RR, 1.23; 95% CI, 1.14–1.33; *P* < 0.001) and progression to PDR (RR, 1.48; 95% CI, 1.20–1.81; *P* < 0.001) associated with smoking in T1D, but a decreased risk of DRD (RR, 0.92; 95% CI, 0.86–0.98; *P* = 0.02) and PDR (RR, 0.68; 95% CI, 0.61–0.74; *P* < 0.001) associated with smoking among people with T2D.

### Other Diabetes Complications as Risk Factors for DRD

Regarding the associations between DRD and other diabetic complications, recently systematically reviewed by Pearce et al,^131^ a lot of the research has focused on DRD as a risk factor rather than as an outcome. For example, DR has been found to be an independent risk factor for CVD in those with diabetes.^131^ For the purpose of the Mary Tyler Moore Vision Initiative DRD Staging Update Project, research with DRD as a dependent variable is considered below.

#### CVD

Few large prospective studies have been specifically designed to evaluate clinical CVD as a prognostic factor of DRD incidence or progression. History of CVD was included as a potential risk factor in the EURODIAB Prospective Complications Study; however, no association (*P* = 0.6) was found to PDR progression in T1D.^132^ A Scottish registry study also found no association (*P* = 0.16) between past CVD events and the development of referable DRD in T1D, and even a negative association (*P* < 0.001) in T2D.^18^ There are more prospective data on peripheral artery disease (PAD) as a separate presentation of CVD. A prospective Taiwanese study reported that PAD, when defined by abnormal ankle-brachial index, was associated with the development of DR (HR, 2.186 [1.261–3.789]; *P* = 0.005) in T2D.^133^ The prospective Action in Diabetes and Vascular Disease: Preterax and Diamicron MR Controlled Evaluation-ON posttrial studying individuals with T2D identified lower-extremity ulceration or amputation at baseline as an independent risk factor (HR, 1.53 [1.01–2.30]; *P* = 0.04) for retinal photocoagulation or diabetes-related blindness.^134^ In a retrospective longitudinal study from the US, nonhealing ulcers (HR, 1.54 [1.15–2.07]; *P* < 0.05) helped to predict progression from NPDR to PDR.^135^

In cross-sectional studies, the findings are conflicting. The presence of coronary heart disease or stroke was independently associated with the presence of DRD (OR, 3.23 [1.09–9.56]; *P* = 0.03) in individuals with T2D from the Cardiovascular Health Study.^136^ A cross-sectional Japanese study on T2D^137^ reported that the presence of coronary artery disease correlated with the severity of DR (*P* < 0.01) and was an independent risk factor for its presence (OR, 1.97 [1.45–2.70]; *P* < 0.01). However, in the Atherosclerosis Risk in Communities cohort, no independent association between coronary heart disease, stroke, or PAD and the severity of DRD could be shown.^34^ A Korean cross-sectional study also found no association between PAD and retinopathy in T2D.^138^

#### Peripheral Diabetic Neuropathy

Regarding peripheral diabetic neuropathy as a risk factor for DRD, few studies were designed to address this question. A retrospective longitudinal study from Taiwan did show an increased risk (HR 5.41 [4.92–5.94], *P* < 0.001) of developing DRD in the presence of diabetic polyneuropathy^139^ and a cross-sectional study from Malaysia identified neuropathy (OR, 2.91 [2.21–3.82]; *P* < 0.001) as a risk factor for prevalent DR in T2D.^140^ A meta-analysis of cross-sectional studies showed that diabetic neuropathy is significantly associated (OR, 1.73 [1.19–2.51]; *P* < 0.01) with DRD.^141^

#### Diabetic Kidney Disease

There are a few prospective studies evaluating the role of diabetic kidney disease (DKD) in DRD. A Taiwanese study reported that diabetic nephropathy was associated with a higher risk of developing NPDR (HR, 5.01; 95% CI, 4.68–5.37) and PDR (HR, 9.70; 95% CI, 8.15–11.5) during follow-up.^142^ In a study from Japan, overt proteinuria and moderately reduced estimated glomerular filtration rate (eGFR) predicted incident treatment-required diabetic eye diseases (HR, 1.91 [1.27–2.87] and HR, 1.90 [1.11–3.23]) and this effect increased (HR, 5.57 [2.40–12.94]) when both were concomitantly present.^143^ In individuals with T1D, a Spanish prospective study has evaluated DKD as a prognostic factor of DRD, finding a significant association only with sight-threatening DRD.^144^ In T2D, prospective data suggest albuminuria (HR, 1.485 [1.103–1.548], *P* < 0.001) to be a better prognostic factor of DR than eGFR (HR, 1.223 [1.098–1.201]; *P* = 0.04), although both are risk factors for DR.^145^ Data from the DCCT show that development of nephropathy independently increases the risk of progression of retinopathy (HR, 1.62 [1.23–2.13]; *P* = 0.001) in T1D.^146^ Furthermore, several cross-sectional studies support the association between DKD and DRD.^141^^,^^147^, ^148^, ^149^, ^150^, ^151^, ^152^

### C-peptide

C-peptide is included here as it is becoming an increasingly recorded measure in the clinical record in T1D. Serum C-peptide level is a measure of residual β-cell function in T1D.^153^ Testing has become more sensitive and cheaper in recent years.^154^ Various methods and assays have been used across studies to measure C-peptide levels. Indeed, C- peptide can be measured in blood levels: either fasting, random nonfasting, or in a formal stimulation test (glucagon stimulation test and mixed-meal tolerance test). C-peptide can also be measured in urine using the urine C-peptide to creatinine ratio. C-peptide levels need to be interpreted with caution in the presence of kidney disease.^153^^,^^154^

Higher levels of C-peptide have generally been found, in several studies, to be negatively associated with prevalent/incident DRD among people with T1D.

A recent study in a representative sample of people with T1D in Scotland (N = 6076) showed that adjusted rates of prevalent and incident DRD were lower in those with nonfasting serum C-peptide ≥ 30 pmol/L versus those with levels < 5 pmol/L and found no evidence of a threshold effect. The model estimates were: OR, 0.69 (95% CI, 0.56–0.85) for the association of C-peptide 30 to 200 versus < 5 pmol/L with odds of DRD at baseline, and 0.66 (0.50–0.89) for incident DRD, adjusting for sex, age at onset, diabetes duration, and HbA1c.^155^ A recent Pittsburgh Epidemiology of Diabetes Complications study^156^ reported that the prevalence of PDR was lower, although not significantly, among those with detectable C-peptide (33.3% vs. 55.1%, *P* = 0.08), in a sample of 185 people with long-duration T1D. Similarly, Kuhtreiber et al^157^ found that fasting C-peptide levels > 10 pmol/L versus ≤ 10 pmol/L was protective against complications, including retinopathy, in people with T1D (N = 324). In those from the DCCT study with intensive treatment, with a short diabetes duration (1–5 years), there were significant reductions in the risk of sustained retinopathy in those with stimulated C-peptide of 0.2 to 0.5 nmol/L compared with those with < 0.2 nmol/L. For a 50% higher stimulated C-peptide on entry, there was a 24.6% (95% CI, 10.7–36.3) risk reduction in sustained retinopathy in unadjusted analyses, 23.8% (95% CI, 8.8–36.3) when adjusting for HbA1c and DRD at entry.^158^ However, the Diabetes Incidence Study in Sweden did not find any evidence of the association between residual C-peptide at onset and the risk of DR 15 years later.^159^

There have also been a few studies investigating the relationship between C-peptide and DRD in people with T2D. The Genetics of Latino Diabetic Retinopathy study among Latinos with T2D found that fasting C-peptide was significantly lower in patients with DR. C-peptide concentration was also inversely associated with the severity of retinopathy (N = 585, β = −0.21; 95% CI [–0.30 to –0.13]). This relationship remained significant after adjusting for confounding factors. These conclusions were supported by similar results obtained on fasting insulin levels.^160^ A study in China (N = 3100) reported that, after adjusting for age, sex, duration of diabetes, BMI, HbA1c, BP, and albuminuria creatinine ratio and insulin treatment, age at diagnosis and postprandial C-peptide (OR, 0.92; 95% CI, 0.86–0.94) were independently associated with DR.^161^ Another study in Italy,^162^ among 931 individuals free from any chronic complications at baseline, found that the risk of incident retinopathy, was negatively associated with the highest C-peptide tertile, after adjusting for age, sex, BMI, smoking, the time since diagnosis, insulin treatment, the HbA1c, systolic BP, HDL cholesterol, and triglyceride values (HR, 0.33; 95% CI, 0.23–0.47). Older studies had previously found no correlation between C-peptide levels and DRD^163^^,^^164^ (See Results S1 for more details, available at www.ophthalmologyscience.org).

Bhatt et al^165^ suggested C-peptide may protect against diabetic vasculopathy, and hence, DRD, highlighting that more studies are needed to understand the cellular mechanisms behind this.

### Pregnancy

Several studies have found pregnancy to be a risk factor for DR beyond that explained by any changes in other known risk factors during pregnancy. As a result, most guidelines recommend intensive screening during pregnancy. In a small study from 1982, pregnant women were found to have a greater cumulative incidence of retinopathy during pregnancy compared with age-similar controls.^166^ Klein et al^167^ found pregnancy associated with a 2.3-fold odds of retinopathy progression. In the DCCT, the odds of ≥3-step progression was 2.9-fold among pregnant versus not pregnant women (*P* = 0.003). The increased odds persisted for 12 months postpregnancy.^168^ Although there are many other studies reporting the incidence of DRD and DRD progression among pregnant women, the above studies are the key ones that have directly compared incidence in comparable cohorts of pregnant and nonpregnant women. Among pregnant women, risk factors for DRD worsening include DRD status prepregnancy, glycemic control, preeclampsia, diabetes duration, and hypertensive and nephropathy status.^169^ More contemporary studies that directly compare incidence or worsening of DRD in pregnant and nonpregnant women adjusting for other risk factors for DRD are lacking; so it remains unclear to what extent pregnancy itself is associated with worsening of DRD currently.

The evidence from the single-factor evaluations presented so far is summarized in Table 1.

### Systemic Inflammation

Two studies have suggested that persons with bacterial infections have an increased risk of DRD progression. One study^135^ using a large US health claims database indicated that persons with nonhealing ulcers had a 54% (1.54 [1.15–2.07]) increased hazard of progressing from NPDR to PDR. Further, analysis of the FinnDiane study subjects revealed that persons with T1D and baseline severe DR had a 1.5 times higher rate of antibiotic purchases and greater lipopolysaccharide activity and had a 1.58 HR for incident DR.^170^ This previously unappreciated association may provide a therapeutic opportunity in some patients.

### Prognostic Models for Incident Retinopathy and Retinopathy Progression in Diabetes

In the previous sections, we summarized findings for 1 risk factor at a time. In this section, we focus on studies that have specifically reported and evaluated multivariable models for DRD onset, progression, and then, in the following section, response to treatment. Our focus is on the overall content and performance of the models and what they can indicate about the importance of considering risk factors together.

We found 13 studies^18^^,^^20^, ^21^, ^22^^,^^171^, ^172^, ^173^, ^174^, ^175^, ^176^, ^177^, ^178^, ^179^ specifically reporting on the development of prognostic models for DRD; 2 studies focused on people with T1D,^172^^,^^173^ 3 studies focused on people with T2D,^177^, ^178^, ^179^ whereas 7 examined a mixture of people with T1D and with T2D.^18^^,^^20^, ^21^, ^22^^,^^171^^,^^174^ Where studies examined several models corresponding to different definitions of DRD, we focused on the model with the widest definition of DRD (i.e., composite outcome). The majority of studies examined the progression of DRD or used a combined outcome measure of DRD onset and progression of DRD. Only 3 studies specifically looked at DRD onset,^20^^,^^172^^,^^177^ with the majority using a composite of retinopathy and maculopathy, and 1 study examined blindness as the outcome.^21^

Most of the models only performed internal validation of the model. The studies that reported on calibration revealed that the models were well calibrated, both in internal and external validation. Discriminatory ability based on the derivation population (internal validation) resulted in C-statistics ranging from 0.55 (95% CI, 0.54–0.56) to 0.92. Of all studies, 6 performed external validation of the model, with most of them showing good performance (range C-statistics: 0.65 [0.61–0.70] to 0.84).

From a prognosis perspective, all 3 studies examining DRD onset found HbA1c and diabetes duration to be significant prognostic factors, while 2 of 3 also retained sex and BP as significant, suggesting that these systemic factors are informative regarding DRD onset.

Among the other studies, 7 used models adjusting for retinal features (ranging from the presence of retinopathy to the number of hard exudates/retinal hemorrhages and color discrimination), and retinal features were retained as a significant prognostic factor of DRD in all the corresponding models. Most of these models also retained the duration of diabetes and glycemic control (HbA1c or glucose level) as significant prognostic factors, showing consistent evidence of a positive association of diabetes duration and glycemic levels with DRD. Of note, glycemic control was expressed using static variables, and more dynamic variables representing the variability of glucose over time were not considered in any of the studies mentioned here. Finally, sex was commonly retained, although to a lesser degree, being considered as a candidate predictor in 12 of the studies and retained in 6. Only 2 of the studies^18^^,^^22^ quantified the increment in prediction resulting from including clinical risk factors apart from retinal information. Scanlon et al^22^ reported area under the curve = 0.774 (95% CI, 0.748–0.800) for a model including grading from 1 screening episode and clinical data, for a model including gradings from 2 screenings only, area under the curve = 0.759 (0.732–0.788), and finally for 2 screenings plus clinical risk factors, area under the curve = 0.786 (0.759–0.813), whereas Ochs et al^18^ reported an increase in C-statistic of 0.013 and 0.016 for prediction of referable DRD in a model including clinical risk factor data beyond a grade. Neither of these studies examined the increment in prediction for each clinical risk factor individually; however, they both showed that the increment of a clinical risk factor apart from retinal grading is minimal.

Our findings also highlighted that factors such as BP, for which there is consistent evidence of strong univariate association with DRD, did not necessarily get retained as significant in a multivariate setting. Indeed, BP was included as a candidate predictor in 10 of the studies but was retained as significant by only 4. Age was included in most of the models but only retained in 3 models, indicating that the duration of diabetes is a stronger prognostic factor for the development of retinopathy compared with age.

Table 2^18^^,^^20^^,^^21^^,^^171^, ^172^, ^173^, ^174^, ^175^, ^176^, ^177^, ^178^, ^179^ summarizes the risk factors that were retained and the significant ones in the final models for each study examined. Any of the factors that were retained in ≥1 models could be said to have reached 1B level of evidence with respect to prognostic use in DRD.

**Table 2:** Prognostic Factors Retained and Found Significant in the Final DRD Model of Interest for Each Study

Outcome	Aspinall Et Al^175^	Aspelund Et Al^171^	Semeraro Et Al^177^∗	Mehlsen Et Al^174^	Tanaka Et Al^178^	Scanlon Et Al^22^	Hippisley-Cox and Coupland Et Al^21^	Lagani Et Al^173^	Kang Et Al^172^∗	Basu Et Al^179^	Dagliati Et Al^176^	García-Fiñana Et Al^20^∗	Ochs Et Al^18^	Times Retained
Factor	Any Retinopathy	PDR or DME	DR	PDR or DME Treatment	DR or DME	DR or DME	Blindness	Worsening DR	NPDR	DR	Specific Lesions	STDR	RDR or RDM	
Age					•		•			•			•	**3**
Sex		•	•				•		•	•			•	**6**
Type of diabetes		•		•			•				•	•		**5**
Deprivation							•							**1**
Ethnicity										•				**1**
Marital status								•						**1**
Postpubescent								•						**1**
Smoking status											•			**1**
BMI								•			•			**2**
Age at diagnosis									•					**1**
Duration of diabetes	•	•	•		•	•	•		•			•	•	**9**
Postprandial blood glucose	•													**1**
Proteinuria	•													**1**
Albuminuria			•											**1**
HbA1c		•	•	•	•	•		•	•	•	•	•	•	**11**
Systolic blood pressure		•	•							•		•		**4**
Cholesterol						•				•			•	**3**
HDL cholesterol										•				**1**
Cholesterol/HDL ratio							•							**1**
Serum creatinine						•				•				**2**
Albumin/creatinine ratio					•					•				**2**
Chronic renal disease							•							**1**
Presence of retinopathy		•				•	•	•					•	**5**
Color discrimination	•													**1**
Hard exudates				•										**1**
Retinal hemorrhages				•										**1**
Appointment attendance												•		**1**
Glucose-lowering medication			•							•				**2**
Antihypertensive medication										•	•			**2**
CVD										•				**1**

BMI = body mass index; CVD = cardiovascular disease; DME = diabetic macular edema; DR = diabetic retinopathy; DRD = diabetic retinal disease; HbA1c = glycated hemoglobin; HDL = high-density lipoprotein; NPDR = nonproliferative diabetic retinopathy; PDR = proliferative diabetic retinopathy; RDM = referable diabetic maculopathy; RDR = referable diabetic retinopathy; STDR = sight-threatening diabetic retinopathy.

∗ Denotes studies with separate models for DR onset.

### Prediction of Therapeutic Responses to Retinal Therapies

Retinal therapies considered in the studies found included panretinal photocoagulation,^180^ anti-VEGF,^181^, ^182^, ^183^, ^184^, ^185^, ^186^, ^187^, ^188^, ^189^, ^190^, ^191^, ^192^, ^193^, ^194^, ^195^, ^196^, ^197^, ^198^, ^199^, ^200^, ^201^, ^202^, ^203^, ^204^, ^205^, ^206^, ^207^, ^208^, ^209^, ^210^, ^211^, ^212^, ^213^ pars plana vitrectomy,^214^ and steroids^215^, ^216^, ^217^ for DME^181^, ^182^, ^183^^,^^185^^,^^187^^,^^188^^,^^191^^,^^192^^,^^194^, ^195^, ^196^, ^197^, ^198^, ^199^, ^200^, ^201^, ^202^, ^203^, ^204^, ^205^, ^206^, ^207^, ^208^^,^^210^^,^^212^, ^213^, ^214^, ^215^^,^^217^^,^^218^ and retinopathy.^180^^,^^219^ Among these studies, the prominent categories for the definition of therapeutic response were either in terms of a change in VA or a change in layer thicknesses within the eye. Our literature search also retrieved studies on age-related macular degeneration and retinal vein occlusion but these were not our focus in this project.

Nearly all studies for DME reported analysis of associations between characteristics at baseline and therapeutic response rather than quantifying predictive value through, for example, the C-statistic. One exception was a study of 127 eyes with DME treated with anti-VEGF injections^205^ in which a deep-learning system to predict change in retinal thickness from OCT images was assessed. The cohort size was small, and the predictive model did not assess increment in predictive performance; however, this study found the system to attain a C-statistic of 0.866. Another exception is a recent study of 712 patients with DME who received anti-VEGF treatment.^192^ Features extracted from an OCT image such as the number of hyper-reflective dots, disruption ratio, and optic density ratio of various retinal layers were combined in a random forest prediction model to predict poor and good responder groups (defined via a decrease in central macular thickness). The reported C-statistic for the model was 0.923 and the sum of hyper-reflective dots was found to be the most relevant feature for prediction.

Factors associated with DME treatment outcomes included the following: baseline HbA1c, DRD severity, mean arterial pressure, cystoid abnormalities near the macula, early VA response, baseline age, baseline visual response, diabetes duration, retinal vein occlusion, change in intraocular pressure, external limiting membrane integrity, absence of surface wrinkling retinopathy, submacular fluid, baseline hyper-reflective foci in the outer retinal layers, baseline subfoveal choroidal thickness, baseline OCT, microRNA-98-5p, number of microaneurysms in deep capillary plexuses, mean arterial BP, and baseline VEGF. The majority of studies had cohort sizes of <250 people. Baseline age and VA were the most consistent associations among larger studies. For example, a study of 502 eyes ranibizumab-treated for DME^190^ found improvement from baseline best-corrected VA letter score of ≥15 correlated with poor baseline best-corrected VA (OR, 0.73; *P* < 0.0001), submacular fluid at baseline (OR, 2.43; *P* = 0.004), shorter duration of diabetes (OR, 0.89; *P* = 0.03), and young age (OR, 0.88; *P* = 0.02). A more recent study of the same trial reached similar conclusions.^207^ In addition, a prospective, multicenter, observational study in Thailand,^212^ considering bevacizumab and ranibizumab treatment for a number of retinal diseases including 1314 eyes with DME, found that having diabetes without other comorbidities was a statistically significant predictor of low response for vision improvement compared with diabetes with other comorbidities (OR, 0.16; 95% CI, 0.03–0.76; *P* = 0.02). The same study found that age and baseline VA score were associated with VA improvement. However, notably, a post hoc analysis of the RIDE and RISE studies including 745 eyes found no association between baseline systemic factors including age, diabetes medication history, HbA1c, eGFR, BMI, BP, and change in VA at 24 months after treatment of ranibizumab for DME.^181^

We have reviewed a large number of clinical risk factors that are routinely captured during the course of diabetes management. There is strong evidence for associations of diabetes duration, HbA1c, and BP with DRD and moderate evidence for age, sex, diabetes type, SES, C-peptide, CVD, eGFR, and pregnancy.

The extent to which SES or its relationship with other DRD risk factors can be modified is an important consideration, as barriers to accessing medical care such as lack of insurance and transportation can be addressed to improve DRD outcomes.

Whether there are ethnic differences in the risk of DRD remains unclear as risks independent of other risk factors have not been consistently found.

There is strong evidence that higher BP is a risk factor for DRD incidence and progression. Clinical trials suggest that BP lowering can reduce the incidence of DRD and, at least in T2D, reduce its progression. However, the ACCORD-EYE study did not find any benefit at lower starting BPs. It should be noted that in the absence of diabetes, hypertension alone may cause morphologic changes in the retinal vessels that are similar to those seen in mild to moderate DRD such as hard exudates, cotton-wool spots, and retinal hemorrhages. Similar to that found in metabolic control, there is no information regarding the effect of the tight control of BP on retinal neurodysfunction or neurodegeneration.

There is clear evidence that having higher average glucose levels, more glycemic variability, and spending less time in the optimal glycemic range are strong risk factors for DRD incidence and progression. While optimizing glucose levels reduces retinopathy incidence, it is unclear whether any specific glucose-lowering agents are particularly beneficial for DRD beyond glycemic control.

The direct effect of the use of these technologies on DRD has not been comprehensively studied, particularly HCL, due to the relatively new nature of this technology. As diabetes treatment technologies such as CSII, CGM, and HCL have a robust effect on glycemic parameters, it is highly likely that their use will lead to a lower risk of DRD. Further studies are needed to examine the effects of newer technologies such as HCL on the incidence and progression of DRD, independent of the effect on improving HbA1c. In particular, reducing glycemic excursions and variability could have benefits beyond improvements in overall hyperglycemia or mean average glucose.

It remains unclear whether lipid-lowering drugs have a substantial impact on DRD. We hypothesize that overall, early studies suggest an important role of lipid-lowering drugs because the higher levels of total cholesterol, low-density lipoprotein cholesterol, and triglycerides were associated with a greater degree of vision loss secondary to increased hard exudate in the macula.^1^,^10^^,^^220^ At the time, treatments for total cholesterol were lacking (i.e., no statins available during ETDRS), whereas the clinical trials with statins took place in an era with better serum lipids and no study was large enough for sufficient power. The fenofibrate trials suggest the treatment effect on DRD may not be mediated through the lowering of lipids, although triglyceride levels were indeed reduced. Based on the results from the FIELD and ACCORD-EYE studies, Wong et al^221^ suggested using fenofibrate as an adjunctive treatment for DRD (while taking into account the risks, costs, and benefits of doing so).

Based on the evidence, there is a plausible biological mechanism through which smoking could contribute to DRD incidence and progression, but mixed evidence in the literature suggests that smoking is not a strong risk factor for DRD, and the association between smoking and DRD may differ between type 1 and T2D.

With respect to diabetes complications as risk factors for DRD, there is a shortage of thorough longitudinal data on whether CVD is an independent risk factor for DRD in individuals with diabetes. However, PAD has been shown to precede DRD in T2D, which is interesting considering the increasing number of studies suggesting neural alterations to be important apart from vascular damage in DRD, a common factor with PAD. An association between diabetic neuropathy and DRD would support this hypothesis, however, the role of diabetic neuropathy as a prognostic factor of DRD needs to be further determined by longitudinal studies. Finally, DKD, a strong driver of other vascular complications, is an independent risk factor for DRD in both T1D and T2D.

Larger-scale studies are needed to generalize findings on C-peptide and DRD in people with T1D and T2D, to better understand the magnitude and shape of this relationship, and to understand how these might vary between specific subgroups (e.g., age, ethnicity). This could also be helpful in evaluating how therapies targeting C-peptide can be beneficial in preventing or delaying the occurrence of diabetic complications, including DRD.

Several prognostic models for the risk of DRD have been developed to estimate the risk of DRD based on individual characteristics. These models contain prognostic factors that are routinely measured in diabetes care, increasing their applicability in clinical practice. Glycosylated hemoglobin and diabetes duration were shown to be consistent predictors of DRD onset, although there was also evidence of associations of BP and sex. Few of these models were evaluated for generalizability or showing good predictive performance in populations beyond the model development population. Most models did not evaluate the marginal improvement in prediction gained beyond retinal imaging itself.

There are a number of studies that provide evidence of the association between systemic factors and response to treatment of DRD and early small-scale studies that estimate the predictive information provided by imaging modalities for predicting the therapeutic response of DME. However, the evidence base for prediction of therapeutic response is not sufficiently consistent for baseline systemic factors and there are currently no large studies that quantify the marginal predictive information of systemic factors within a multivariate model. Furthermore, no such predictive model has been externally validated across diverse cohorts.

### Gap Analysis and Limitations

The gap analysis reported that apart from diabetes duration and sex, further data are needed to clarify the role of all the above individual risk factors in DRD incidence and progression. Glycemia was found to have a very clear association with DRD, as was hypertension. However, even for these systemic factors, further data are required to precisely quantify the incremental predictive improvement to prognosis and prediction provided by parameters derived from continuous glucose monitors and from ambulatory BP monitors. Pregnancy was consistently reported to be associated with retinopathy worsening, though recent studies reflecting modern care are lacking.

Larger data sets are needed to resolve what prognostic models of retinopathy have the best performance. Furthermore, calibration of models to the prevailing disease incidence levels in any given location or setting will always be required.

Properly validated models of prediction of therapeutic response for anti-VEGF and laser therapy do not exist. Overall, because the focus here is on the value of systemic factors, the fact that only a small number of modeling efforts have tried to quantify prognosis/prediction beyond retinal measures attests to the need for a wider effort to develop and internationally validate retinopathy prognosis and prediction models that evaluate systemic factors apart from the retinal imaging.

Limitations of the work presented here include the pragmatic approach adopted for reviewing individual risk factors.

### Discussion

Our review highlights that many risk factors for which data exist in clinical records routinely show strong associations with DRD incidence or progression, or both, in observational cohort studies. There is consistent evidence for associations of diabetes duration, HbA1c, and BP with DRD. In particular, there is strong trial evidence for HbA1c and systematic reviews of trials support an effect of BP lowering at higher starting BPs.

Nevertheless, data pertaining to more precise measures of variability such as CGM and ambulatory BP monitoring measurements are needed to better understand the relationship of these parameters with DRD. It is unclear whether there are associations between SES, ethnicity, and DRD beyond associations of these with risk factors of DRD.

More sporadic evidence has been observed for associations of factors such as lipids, or other diabetes complications with DRD. For pregnancy, studies consistently found worsening but the extent to which contemporary pregnancy care has reduced that risk is unclear.

However, with respect to our grading and prognostic goals, what is ultimately necessary is not more studies of each individual potential risk factor but data on the marginal increment in prognostic information provided by each risk factor within the context of a multivariate model. In multivariate models, HbA1c and diabetes duration were consistently retained as prognostic factors of DRD onset.

Most studies examine the prognosis of DRD progression alone or combined with DRD onset as a composite outcome adjusted for retinal features. However, evidence for the marginal increment in prognostic information gained by including such systemic factor data apart from retinal measures is sparse. In the 2 studies that did evaluate this, retinal imaging was found to be by far the strongest predictor of DRD, and there was no useful increment in prognostic information resulting from the inclusion of systemic factors. Large-scale studies of the increment in prognostic information gained by such data apart from any existing or new imaging or multivariate staging system (including retina physiology, visual function, and quality of life variables) are needed.

Furthermore, at present, personalized medicine is not feasible because refinement of the prediction of response to treatment for DRD is badly needed. One might envisage using such data apart from imaging for personalized prediction of retinopathy risk or progression and informing personalized screening intervals.

Cost and ease of availability of factors of interest will need to be taken into account. However, even with an improved quantification of marginal predictive and prognostic information of risk factors with respect to multivariate models that include information from imaging and other modalities, it is important to keep in mind that such models are also quickly becoming out-of-date with; for example, the introduction of new imaging techniques compared with the 2-dimensional ETDRS-style grading system or novel ways of processing images from existing modalities becoming the new standard (e.g., deep learning).

We therefore need an international network of centers able to draw on data from large diverse cohorts to quickly and efficiently evaluate the prognostic and predictive benefit of risk factors and imaging technologies in light of these rapid developments.

## Supplementary Data

Box S1
