# Supplementary material for: Role of Systemic Factors in Improving the Prognosis of Diabetic Retinal Disease and Predicting Response to Diabetic Retinopathy Treatment
Source: Ophthalmol Sci. 2024 Feb 17;4(4):100494. doi: 10.1016/j.xops.2024.100494 (PMC11061755; doi:10.1016/j.xops.2024.100494)
Supplement: Box S1 [file mmc1.docx]

**Box 1. Systemic Factors Examined for Association with Diabetic Retinal Disease**

- Age
- Sex
- Diabetes duration
- Diabetes type
- Socioeconomic status
- Ethnicity
- Glycemia/HbA1c
- Glycemic variability
- Blood pressure/ Hypertension
- BMI
- Insulin resistance
- Lipids/ Triglycerides and fenofibrate
- Smoking
- Current diabetes treatments and modalities i.e. pumps and CGM Technology
- Other diabetes complications
- C-peptide
- Pregnancy

Abbreviations: HbA1c: glycated hemoglobin; BMI: body mass index; CGM: continuous glucose monitoring.
